# Completion surgery vs. primary TME for early rectal cancer: a national study

**DOI:** 10.1007/s00384-021-04083-6

**Published:** 2021-12-16

**Authors:** William J. Lossius, Tore Stornes, Tor A. Myklebust, Birger H. Endreseth, Arne Wibe

**Affiliations:** 1 Department of Surgery, St. Olavs Hospital, Trondheim University Hospital, Pb 3250 Torgarden, 7006 Trondheim, NO Norway; 2 Department of Registration, Cancer Registry of Norway, Oslo, Norway; 3 Department of Research and Innovation, Moere and Romsdal Hospital Trust, Aalesund, Norway; 4 Institute of Clinical and Molecular Medicine, Norwegian University of Science and Technology, Trondheim, Norway

**Keywords:** Early-stage rectal cancer, TEM, TAMIS, Survival, Completion surgery

## Abstract

**Purpose:**

While local excision by transanal endoscopic microsurgery (TEM) or transanal minimally invasive surgery (TAMIS) is an option for low-risk early rectal cancers, inaccuracies in preoperative staging may be revealed only upon histopathological evaluation of the resected specimen, demanding completion surgery (CS) by formal resection. The aim of this study was to evaluate the results of CS in a national cohort.

**Method:**

This was a retrospective analysis of national registry data, identifying and comparing all Norwegian patients who, without prior radiochemotherapy, underwent local excision by TEM or TAMIS and subsequent CS, or a primary total mesorectal excision (pTME), for early rectal cancer during 2000–2017. Primary endpoints were 5-year overall and disease-free survival, 5-year local and distant recurrence, and the rate of R0 resection at completion surgery. The secondary endpoint was the rate of permanent stoma.

**Results:**

Forty-nine patients received CS, and 1098 underwent pTME. There was no difference in overall survival (OR 0.73, 95% CI 0.27–2.01), disease-free survival (OR 0.72, 95% CI 0.32–1.63), local recurrence (OR 1.08, 95% CI 0.14–8.27) or distant recurrence (OR 0.67, 95% CI 0.21–2.18). In the CS group, 53% had a permanent stoma vs. 32% in the pTME group (*P* = 0.002); however, the difference was not significant when adjusted for age, sex, and tumor level (OR 2.17, 0.95–5.02).

**Conclusions:**

Oncological results were similar in the two groups. However, there may be an increased risk for a permanent stoma in the CS group.

## Introduction

Local excision (LE) is an appealing solution in early-stage rectal cancer (ERC), facilitating organ preservation, implying less postoperative morbidity and mortality, and offering better functional outcomes as compared with rectal resection [1–3]. There is no clear definition of ERC, but for T1 tumors that can be safely removed with free margins and with a low risk of lymph node metastases, a local excision may be considered [4–7]. According to Norwegian guidelines, local excisions of ERC may be performed by transanal endoscopic microsurgery (TEM) or transanal minimally invasive surgery (TAMIS) for patients with superficial (sm1) T1 tumors, < 3 cm in diameter, without high-risk histopathological features like poor differentiation, tumor budding, or lymphovascular invasion [8]. In this setting, although a higher rate of local recurrence has been demonstrated, overall survival has been comparable after TEM and TME [9].

Radiochemotherapy has no role in the treatment of ERC in Norway, except postoperatively in case of tumor infiltrating resection margins.

Conventional transanal excision has proven inferior to TEM with regard to the quality of resection (surgical margins, fragmentation of the specimen) and local recurrence [10, 11], and in Norway this technique has been replaced by TEM or transanal minimally invasive surgery (TAMIS), two techniques reported to have comparable outcomes [12].

Preoperative staging of ERC is a major clinical challenge. Regarding T-stage, a meta-analysis on MRI demonstrated a sensitivity of 87% and a specificity of 75%, and for N-stage, the rates were 71% and 77%, respectively [13]. For endoscopic ultrasound (EUS), a sensitivity of 88% and a specificity of 98% on staging T1 cancers have been reported [14]. However, in two recent reports on EUS staging, one reported inaccurate T-staging in 44.8% of the cases and another reported the accuracy for T1 and T2 stages as 66% and 57%, respectively [15, 16]. The inaccuracies in preoperative staging, despite the use of EUS and MRI, will result in surgeons inadvertently performing local excision with TEM or TAMIS on high-risk early rectal cancer (i.e., cancers with invasion beyond T1sm1 or other high-risk features). Late recurrence after TEM is associated with reduced survival, particularly due to increased risk of metastases [17]. Hence, the recommended strategy for inadvertently locally excised high-risk early rectal cancer, i.e., revelation of high-risk histopathological features upon examination of the local excision specimen, has been completion surgery (CS) with TME within a few weeks [8]. Although evidence is scarce, a few studies have reported that oncological outcomes after LE and CS are comparable to those following primary TME surgery [18–21].

The number of patients with ERC has increased following implementation of colorectal cancer (CRC) screening programs, and knowledge of the outcomes of this group of patients is increasingly important.

The primary aim of this study was to evaluate oncological outcomes of completion surgery compared with primary TME surgery for ERC in a national cohort. A secondary aim was to evaluate the rate of permanent stoma formation at CS vs. primary TME for ERC.

## Methods

The Norwegian Colorectal Cancer Registry (NCCR) is part of the Cancer Registry of Norway. The NCCR prospectively collects data on all colorectal cancers in Norway and has an estimated completeness of 99% [22]. Clinicians involved in the treatment of CRC are committed to reporting data on work-up, treatment, and follow-up. From this registry, the present cohort included all patients diagnosed with T1 and T2 rectal cancer in the period 2000–2017, first treated with a local resection with TEM or TAMIS and later completion surgery (CS) by TME in a population-based retrospective study. For comparison, the control group consisted of all patients with T1 and T2 tumors without distant metastases and/or neoadjuvant therapy undergoing primary TME surgery in the same period. The time period was chosen to allow for an adequate sample size and follow-up time. The NCCR defines a local recurrence as a recurrence in the pelvis, either clinical or histopathologically verified, occurring later than 4 months after primary resection. Hence, we have defined TME surgery within 4 months of a primary LE as a completion procedure. A distant recurrence occurring within 4 months of primary surgery was considered a synchronous metastasis. Rectal cancer was defined as a tumor within 16 cm from the anal verge measured on a rigid proctoscope. Possible confounding variables were age, sex, tumor level, T-stage including sm-classification for local excisions, N-stage, differentiation, and type of procedure.

Categorical variables were described as frequencies and compared by Pearson’s chi-square test and Fisher’s exact test, where appropriate. Continuous variables were summarized as means and compared by independent sample *t*-test. For survival analyses, individuals were followed from the date of surgery (completion surgery or primary TME) until the date of the event of interest (death from all causes, local recurrence or metachronous metastasis) or the date of administrative censoring (Dec 31, 2018 for CS and Dec 31, 2019 for primary TME), whichever came first. When analyzing the risk of local recurrence and metachronous metastases, individuals who died were censored at the date of death. Unadjusted survival curves were estimated by the Kaplan–Meier method and differences analyzed by the log-rank test. Multivariable Cox proportional hazard regressions were used to estimate hazard ratios (HRs) of CS vs. primary TME, adjusted for relevant confounders. Univariable and multivariable logistic regressions were used to estimate odds ratios (ORs) of permanent stoma, again comparing CS with primary TME. To examine the robustness of the results from multivariable regressions we also performed propensity score matched analyses using the psmatch2 command in Stata, and including the same set of potentially confounding variables, as in the standard multivariable regressions, as the basis for calculating propensity scores. After calculating propensity scores, Cox and logistic regressions were fitted with the case–control indicator and the propensity score as the only covariates. *P*-values < 0.05 were considered significant. Statistical analyses were performed with IBM SPSS version 23 for Windows and Stata version 17.0.

## Results

There were 49 patients with T1–T2 tumors without distant metastases treated by completion surgery after local excision by TEM or TAMIS for ERC in the period 2000–2017 and 1140 patients undergoing primary TME for ERC in the same period. No patients in the CS group received neoadjuvant radiochemotherapy, and 42 patients were excluded from the control group based on neoadjuvant radiochemotherapy, resulting in a control group of 1098. Local excision was performed by TEM in 46 patients and by TAMIS in 3 patients. There was a significant difference in the mean tumor level between the two groups, 6.5 (SD 3.6) cm in the CS-group compared with 8.5 (SD 4.3) in the control group. The mean tumor size was 22 mm (SD 12 mm). The remaining baseline characteristics are given in Table 1.

**Table 1:** Baseline characteristics

Variables		CS	P-TME	*P*-value
Age (mean)		65.0 (+ / − 8.8)	69.0 (+ / − 11.4)	0.02
Sex (male %)		53.0	55.6	0.73
Tumor level (mean cm)		6.5 (+ / − 3.6)	8.5 (+ / − 4.3)	0.002
pT-stage				0.000
	T1	27 (55.10%)	270 (24.59%)	
	T2	22 (44.90%)	828 (75.41%)	
pN-stage				0.56
	N0	38 (77.55%)	889 (80.97%)	
	N1	10 (20.41%)	169 (15.39%)	
	N2	1 (2.04%)	40 (3.64%)	
Differentiation				0.31
	High/moderate	47 (95.92%)	1009 (92.89%)	
	Low	2 (4.08%))	89 (8.11%)	
Type of procedure				0.001
	APR	25 (51.02%)	294 (26.78%)	
	LAR	23 (46.94%)	745 (67.85%)	
	Hartmann	1 (2.04%)	59 (5.37%)	
Resection margin				
	R0	49 (100%)	1072 (97.63%)	
	R1/2	0 (0%)	26 (2.36%)	

*CS* completion surgery. *P-TME* primary total mesorectal excision. *APR* abdominoperineal resection. *LAR* low anterior resection

The indications for completion surgery were T1Sm3 or T2 in 39 patients, and 10 patients had other adverse histopathological features, including non-radical resection margins, poor differentiation, tumor budding, or lymphovascular infiltration. The mean time between LE and CS was 5.7 weeks (SD 2.9, range 1–14). Two patients had remnants of adenocarcinoma exclusively in the bowel wall on the completion surgery specimen, 10 patients had malignant nodes without remnants of adenocarcinoma in the bowel wall, and 1 patient had both residual tumor and malignant nodes, for a total of 13 patients (26.5%) with residual malignancy at the time of completion surgery. All the patients had a radical resection at completion surgery.

**Fig. 1:**
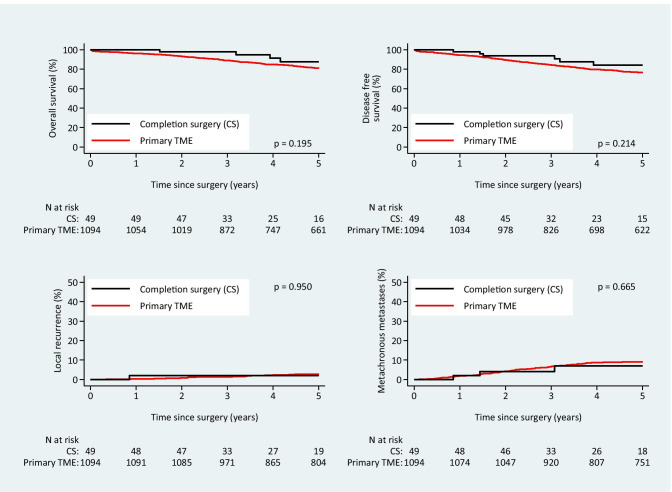
Kaplan–Meier plots illustrating overall survival, disease-free survival, local recurrence, and metachronous metastases of completion surgery compared with primary total mesorectal excision

The 5-year overall survival was 87.5% (95% CI 69.3–95.2) in the CS group and 81.0% (95% CI 78.4 – 83.3) in the primary TME group (*P* = 0.20), and 5-year disease-free survival was 84.2% (95% CI 67.4–92.8) and 76.6% (95% CI 73.8–79.1), respectively (*P* = 0.21). The 5-year local recurrence rate was 2.0% (95% CI 0.3–13.6) in the CS group compared with 2.8% (95% CI 2.0–4.1) in the primary TME group (*P* = 0.95), and the 5-year distal recurrence was 7.1% (95% CI 2.3–20.8) and 9.1% (95% CI 7.5–11.0) in the same groups, respectively (*P* = 0.67). Kaplan–Meier survival curves are presented in Fig. 1. There was no significant difference in overall survival, disease-free survival, local recurrence or distant recurrence by multivariable or propensity score matched analyses (Table 2).

**Table 2:** Multivariable and propensity score analyses of oncological outcomes

	**Overall survival**		**Disease-free survival**		**Local recurrence**		**Distant recurrence**	
	HR (95% CI)	*P*	HR (95% CI)	*P*	HR (95% CI)	*P*	HR (95% CI)	*P*
P-TME	1		1		1		1	
CS	0.73 (0.27–2.01)	0.55	0.72 (0.32–1.63)	0.43	1.08 (0.14–8.27)	0.93	0.67 (0.21–2.18)	0.51
Tumor level	1.01 (0.96–1.06)	0.72	1.00 (0.96–1. 04)	1.00	0.94 (0.82–1.06)	0.31	0.99 (0.92–1.06)	0.77
Age	1.07 (1.05–1.08)	0.000	1.04 (1.02–1. 05)	0.000	0.97 (0.94–1.05)	0.10	0.98 (0.97–1.00)	0.067
Female	1		1		1		1	
Male	1.75 (1.30–2.36)	0.000	1.62 (1.25–2.12)	0.000	1.10 (0.52–2.32)	0.80	1.13 (0.76–1.70)	0.55
pN0	1		1		1		1	
pN1	1.90 (1.35–2.67)	0.000	2.14 (1.59–2.87)	0.000	3.45 (1.53–7.78)	0.003	3.20 (2.05–4.99)	0.000
pN2	2.65 (1.51–4.67)	0.001	2.62 (1.50–4.24)	0.000	5.10 (1.66–15.7)	0.004	4.93 (2.58–9.42)	0.000
pT1	1		1		1		1	
pT2	1.14 (0.78–1.63)	0.47	1.24 (0.90–1.71)	0.19	4.21 (0.98–18.0)	0.05	1.27 (0.75–2.14)	0.37
LAR	1		1		1		1	
APR	1.20 (0.75–1.91)	0.44	1.23 (0.82–1.85)	0.31	1.05 (0.34–3.30)	0.93	1.51 (0.81–2.84)	0.20
Hartmann	2.34 (1.43–3.85)	0.001	2.17 (1.35–3.49)	0.001	1.74 (0.65–8.56)	0.49	0.82 ( 0.25–2.77)	0.75
HM diff	1		1		1		1	
Poor diff	1.27 (0.79–2.03)	0.32	1.36 (0.91–2.03)	0.13	0.87 (0.25–2.94)	0.82	2.28 (1.37–3.80)	0.001
PSM P-TME	1		1		1		1	
CS	0.70 (0.26–1.90)	0.49	0.72 (0.33–1.71)	0.50	1.10 (0.14–8.40)	0.93	0.82 (0.25–2.65)	0.74

*P-TME* Primary TME. *CS* completion surgery. *LAR* low anterior resection. *APR* abdominoperineal resection. *HM* high or moderate differentiation. *PSM* propensity score matched

Fifty-three per cent of the patients in the CS group received a permanent stoma compared with 32% in the primary TME group (*P* = 0.002), with a two-fold risk of stoma formation (*P* = 0.003); however, the difference was no longer significant when adjusted for tumor level, age, and sex (*P* = 0.07) or by propensity score matched analysis (*P* = 0.13) (Table 3).

**Table 3:** Analysis of permanent stoma formation

		**Permanent stoma**	
		HR (95% CI)	*P*
**Unadjusted**	P-TME	1	
	CS	2.41 (1.36–4.29)	0.003
**Adjusted**	P-TME	1	
	CS	2.17 (0.94–5.02)	0.07
	Tumor level	0.51 (0.48–0.56)	0.000
	Female	1	
	Male	1.21 (0.84–1.75)	0.30
	Age	1.05 (1.03–1.06)	0.000
**PSM**	P-TME	1	
	CS	1.74 (0.85–3.56)	0.13

*P-TME* primary TME. *CS* completion surgery. *PSM* propensity score matched

## Discussion

The main findings in this study were that 5-year disease-free and overall survival as well as the rates of local and distant recurrences after TEM or TAMIS and completion surgery were comparable with those of primary TME surgery for ERC. However, more than half of the CS patients needed a permanent stoma, compared with one-third of the patients of the TME group.

Due to a limited number of cases in which completion surgery is indicated, there are no large studies on the oncological outcomes of CS. Moreover, the heterogeneity of the cohorts within many earlier studies does not allow for a direct comparison of results. This study reports the outcomes of everyday clinical practice at a national level and attempts to provide information before making a choice of treatment when facing early rectal cancer. The low accuracy of preoperative T-staging is a major clinical problem, also reflected in the present cohort, as nearly half of the patients undergoing CS had a T2 tumor, despite the use of both MRI and EUS are recommended as part of the preoperative workup in the national guidelines. Interestingly, only three out of 49 patients had tumor remnants in the bowel wall in the specimen after CS, and all the patients had a radical resection at completion surgery. Including patients with malignant lymph nodes in the specimen, one out of four in the completion group had residual disease, justifying the need for completion surgery in high-risk ERC. In comparison, Jones et al. reported residual disease in 39% of the patients in their review [18].

The high number of APRs in the completion group is a matter of concern. However, the significant difference in tumor level between the two groups (6.5 cm vs. 8.5 cm) may suggest that the lower located tumors of the completion surgery patients put them at increased risk of non-sphincter preserving surgery even before undertaking the TEM or TAMIS procedure. This is also reflected in the multivariable analysis, where the risk of permanent stoma formation was no longer strictly significantly different between the CS and primary TME groups when adjusted for tumor level, age, and sex. However, there was still a clear tendency, and one might argue that a larger sample would have shown significant differences. This may be a result of full-wall TEM resections in the lower rectum causing some fibrotic scarring and, consequently, challenges conducting a safe LAR procedure in this group. Completion surgery may be difficult, and as reported by Hompes et al. the quality of the specimen may be poorer [19], and the stoma rate may be higher. In order to avoid such problems, submucosal dissection, either by TEM or by endoscopic submucosal dissection (ESD), has been advocated [23]. Local excisions are performed on the presumption of facing either an advanced adenoma or a low risk early rectal cancer that can be cured by local excision. If the histopathology report after local excision excludes malignancy, the choice of a technique, either submucosal or full wall thickness, makes no difference. One may argue that submucosal dissection, in preserving the integrity of the muscular wall of the rectum, negates the difficulties on completion surgery described by Hompes. On the other hand, one loses the opportunity to treat some early rectal cancers with local excision alone, as partial thickness excision may be oncologically inadequate. Furthermore, submucosal dissection will not allow for reliable grading of submucosal invasion, resulting in possible T1sm1 cancers being overtreated with completion surgery.

While the present results indicate that there is no oncological loss from performing local excision before completion surgery, they may indicate that local excisions alter the completion TME from LAR to APR, and a particular discussion about this outcome should be held with patients before any strategy is chosen, especially in patients where the tumor level dictates that a primary low anterior resection is possible with 1–2 cm of distal margin. This must be weighed against the probability of local excision actually offering final treatment in correctly staged tumors. Patients with low early rectal cancers where APR would be the primary TME procedure may be especially suited for local excision, as any discussion on the increased risk of stoma formation is not relevant to these patients.

The mean time for CS in this study was 5.7 weeks, and the results indicate that this was within a proper time frame, as advocated by others who have recommended completion within 6 weeks or when the wound after local excision has healed [19].

## Strengths and limitations

The strength of this study relies on histopathological and surgical data and oncological outcomes, which were extracted from a national cancer database with high completeness. The cohort included all patients in Norway treated with completion surgery from a period of 18 years, reflecting everyday practice, and no patients received radiotherapy or chemotherapy.

The limitations lie in the asymmetrical sizes of the two groups, which may allow for a hidden selection bias, like the subjective assessment of the surgeon when suggesting a local resection. The only way to eliminate such hidden bias is by a randomized controlled trial, which we believe would face significant inclusion rate challenges, and thus, the present results were supported by adjusting for the most common objective confounding factors.

## Conclusions

The rates of local and distant recurrences as well as the 5-year overall and disease-free survival were similar in patients receiving completion surgery or primary TME for ERC. However, completion surgery was associated with a higher rate of APR compared with primary TME. The present data support the practice of local excision supported by possible completion surgery from an oncological perspective, but the high rate of APR is of concern. Further studies on large cohorts are required to give definitive recommendations for patients presenting with early rectal cancer.

## Data Availability

Stored locally according to institutional regulations. The study was approved by the regional ethics committee (2010/149.2).
